# Sodium Alginate/Pectin/Gelatin-Based Biopolymer Films Fortified with Pomegranate Peel Extract

**DOI:** 10.1021/acsomega.6c00322

**Published:** 2026-06-11

**Authors:** Ibrahim Yazgac, Bugra Ocak, Gulsah Turkmen

**Affiliations:** † 37509 Ege University, Graduate School of Natural and Applied Sciences, Materials Science and Engineering, 35040 Bornova, Izmir, Turkiye; ‡ Faculty of Engineering, Department of Leather Engineering, Ege University, 35040 Bornova, Izmir, Turkiye

## Abstract

For the fabrication of advanced packaging matrices with enhanced barrier performance and extended product shelf life, bioderived materials offer sustainable alternatives to conventional nonbiodegradable polymers. This study aimed to fabricate and systematically evaluate sodium alginate (SA)/pectin (P)/gelatin (G) films incorporated with 0–2% (w/w) pomegranate peel extract (PPE) with respect to their mechanical, physical, barrier, thermal, and morphological characteristics. A significant (*p* ≤ 0.05) enhancement in thickness, tensile strength (TS), and elastic modulus (EM) was observed in SA/P/G-based films with increasing concentrations of PPE. Conversely, increasing the concentration of PPE in the films led to a significant (*p* ≤ 0.05) and progressive reduction in moisture content (MC, %), water solubility (WS, %), water vapor permeability (WVP), elongation at break (EAB, %), and light transmittance across all formulations. Incorporation of 0.5–2% PPE into SA/P/G films enhanced thermal stability as shown by DSC, improved cross-sectional compactness observed via SEM, and significantly increased DPPH and ABTS radical-scavenging capacities to 85.64 and 78.76% through protein–polysaccharide–polyphenol interactions. The study demonstrated that SA/P/G-based films incorporating PPE represent a viable eco-friendly alternative to conventional packaging materials.

## Graphical abstract

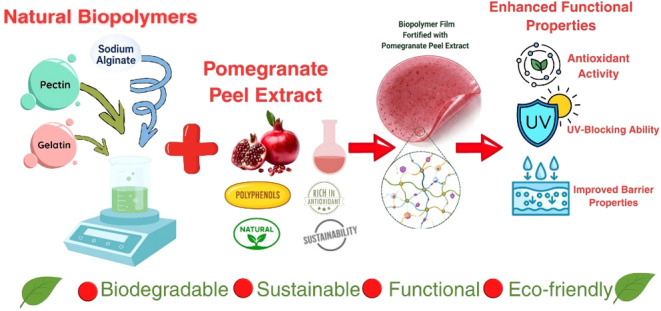

## 1 Introduction

Serving to protect products from environmental contamination, physical damage, thermal and light exposure, and microbial degradation, packaging technologies play a critical role in product preservation because of their broad applicability, versatility, and ease of implementation. The environmental and safety risks of petroleum-based plastics, which account for over 40% of global plastic waste, have driven interest in developing biodegradable and functional biopolymer-based food packaging materials, such as polysaccharides and proteins, offering a sustainable platform for demand-driven active packaging films.^1–3^ Naturally derived biopolymers, such as polysaccharides and proteins, are ideal for replacing conventional plastic films due to their edibility and biodegradability, but their high hydrophilicity makes them prone to water absorption, swelling, and poor barrier and mechanical properties.^1,4^

Polymer blending has proven to be a promising strategy for producing biopolymer films with enhanced functional properties, as combining protein and polysaccharide polymers enables the formation of novel polymeric structures through electrostatic and hydrogen bonding interactions, although overly high proportions of either component can lead to phase separation and aggregation.^5–7^ Sodium alginate (SA) is a linear, anionic, water-soluble polysaccharide derived from brown algae, composed of α-L-guluronic (G) and β-D-mannuronic (M) residues linked by 1–4 glycosidic bonds, and, owing to its abundant carboxylate (−COO^–^) groups that confer polyelectrolyte behavior in aqueous media, it is widely employed in research and industrial film and coating applications due to its biocompatibility, functional properties, and film-forming capacity.^3,8,9^ Although SA can interact with anthocyanins through hydrogen bonding and electrostatic forces, the inherent brittleness of SA films limits their standalone application in biodegradable packaging by impairing polymer chain mobility, reducing flexibility and mechanical strength, and compromising overall material stability.^9,10^ Rich in hydroxyl, carboxyl, and amino functional groups and obtainable from bovine, porcine, or fish sources, gelatin (G)—a nontoxic biopolymer of bioactive polypeptides derived from collagen in animal hides, bones, and connective tissues—represents a prominent candidate among biopolymers.^5,7,11^ G has garnered significant interest due to its film-forming capacity, high availability, transparency, biocompatibility, and potential as a carrier for diverse compounds and additives.^5,12^ Providing protection to packaged products against oxidative damage, the triple-helix structure of G imparts physical strength and UV absorption.^6,7^ However, G, a widely used biopolymer in packaging films, exhibits inherent hydrophilicity and brittleness, limiting its direct application, an issue that can be mitigated by blending it with natural ionic polysaccharides.^11–14^ Pectin (P), a naturally occurring, water-soluble negatively charged polysaccharide primarily consisting of α-(1→4)-D-galacturonic acid and typically extracted from fruit and vegetable residues, including citrus peels and apple pomace, is considered a promising film-producing polymer due to its biodegradability, biocompatibility, ability to form continuous films, superior gas barrier performance, and capacity to prevent lipid migration and water evaporation.^1,9^ However, P-based films exhibit limited mechanical strength and typically require blending with proteins or other polysaccharides to improve their functional properties.^1,9^

Pomegranate (*Punica granatum* L.), a nutritionally rich and bioactive fruit native to the Middle East and Mediterranean region, is now widely cultivated in tropical and subtropical areas and consumed worldwide in forms such as juice and jam, with rising popularity in Western countries due to its high antioxidant and dietary value.^15–17^ Pomegranate peel, making up 40–50% of the fruit and often discarded during juice, wine, and other processing, adds to global food waste—nearly 1.3 billion tons yearly—prompting the United Nations to target a 50% reduction by 2030.^18,19^ Pomegranate peel, rich in pectin, cellulose, hemicellulose, phenolics, anthocyanins, and hydrolyzable tannins, contains antioxidant, antimicrobial, and anti-inflammatory compounds, making it a low-cost, sustainable resource for biodegradable films, yet it is often discarded, contributing to waste instead of being repurposed for innovative applications such as packaging materials.^20–22^ Due to its functional properties, PPE has been widely incorporated into various biopolymer films, including carboxymethyl cellulose/gelatin,^23,24^ polylactic acid,^25^ gelatin/cress seed gum,^26^ and fish gelatin-based films,^15^ for active food packaging applications.

The aim of this study is to develop a novel and sustainable ternary SA/P/G biocomposite matrix for active food packaging applications, addressing the structural and functional limitations often associated with single or binary biopolymer systems. Unlike many previously reported active film design methods requiring nanoparticles, chemical cross-linkers, or synthetic reinforcing agents, the present study developed a ternary film system using only biopolymer-based materials, gelatin, pectin, sodium alginate, glycerol, and pomegranate peel extract. By incorporating PPE—reutilized from pomegranate peel waste—into this specific hybrid framework, the research not only promotes a circular economy by preventing landfill disposal but also uniquely investigates the synergistic compatibility of its bioactive compounds within a complex ternary film structure. Distinguishing itself from existing literature, this study employs a systematic two-step optimization strategy: first, the water barrier properties of the SA/P/G composite films were rigorously evaluated by varying the G content to establish an ideal base formulation; subsequently, this optimized matrix was enriched with four different PPE concentrations. The resulting active films were comprehensively analyzed for their physical, mechanical, antioxidant, and barrier properties and further characterized using FTIR, SEM, and DSC to elucidate the molecular interactions and justify the work’s specific contribution to the field of advanced biodegradable packaging.

## 2 Materials and Methods

### 2.1 Materials

Distilled water was used throughout all stages of solution preparation, and sodium alginate (powder), pectin (from citrus peel, galacturonic acid ≥ 74.0%, powder), gelatin (from bovine hide), 2,2-diphenyl-1-picrylhydrazyl (DPPH), 2,2′-azino-bis-(3-ethylbenzo-thiozoline-6-sulfonic acid) diammonium salt (ABTS), and glycerol (≥99.5%) were purchased from Sigma–Aldrich (Germany).

### 2.2 Methods

#### 2.2.1 Pomegranate Peel Extraction and Characterization

Pomegranates obtained from a local market were peeled, and the resulting peels were washed with tap water, disinfected in 1% (w/w) sodium hypochlorite solution, dried in an oven at 40 °C for 24 h, and milled into powder with a blender. Extraction was carried out at room temperature overnight with stirring, using 20 mL of distilled water per gram of PP powder. The PPE was filtered through Whatman paper to remove insoluble matter, lyophilized for drying, and stored at −18 °C in the dark. The PPE, containing 7.70 ± 0.39% moisture and 80.58 mg gallic acid equivalents of total phenolics per gram of peel powder, was used to prepare SA30/P30/G40-based films.

#### 2.2.2 Preparation of SA/P/G Films and SA/P/G/PPE-Based Films

A series of preliminary tests were conducted to determine the optimal SA/P/G film blend ratio with the best water barrier properties. For this purpose, four different film-forming formulations were prepared. Table 1 illustrates the preparation of SA/P/G composite films with different ratios using the casting technique while keeping the total biopolymer content constant at 5% (w/v). The four prepared film-forming solutions were designated as SA40/P40/G20, SA35/P35/G30, SA30/P30/G40, and SA25/P25/G50, respectively.

**Table 1:** Composition of Film Formulations

film samples	SA (gr)	P (gr)	G (gr)
SA40/P40/G20	2.0	2.0	1.0
SA35/P35/G30	1.75	1.75	1.5
SA30/P30/G40	1.5	1.5	2.0
SA25/P25/G50	1.25	1.25	2.5

To achieve this, SA, P, and G were combined in varying proportions in deionized water with magnetic stirring, with the SA solution obtained by dissolving 1.25–2.0 g of SA powder in 100 mL of distilled water with continuous stirring at 900 rpm in a water bath maintained at 40 °C for 1 h. P powder (1.25–2.0 g) was dispersed in 100 mL of distilled water under continuous stirring on a hot plate at 70 °C for 45 min to obtain a uniform solution, while the G solution (1.0–2.5 g) was obtained by dissolving G in distilled water at 40 °C with continuous stirring for 1 h. The prepared SA, P, and G solutions were thoroughly stirred for at least 1 h to obtain homogeneous mixtures at different mass ratios, as shown in Table 1. Subsequently, glycerol was incorporated as a plasticizing agent at 30% (w/w, relative to the total SA/P/G weight), and the mixture was stirred for a further 1 h, yielding a homogeneous film-casting solution.

The formulation exhibiting optimal water barrier properties, defined by the lowest moisture content (MC, %), water solubility (WS, %), and water vapor permeability (WVP), was selected, and specified amounts of PPE (0.5–2.0% w/w relative to the total SA/P/G weight) were added and stirred for 60 min to prepare SA/P/G/PPE-based films with varying PPE concentrations. Finally, a 25 mL portion of the prepared solution was poured into Petri dishes with a diameter of 12 cm, dehydrated at 45 °C for 36 h, and the resulting films were removed and stored in a desiccator maintained at 25 °C and 50% relative humidity for subsequent analyses.

#### 2.2.3 Film Thickness and Mechanical Properties

Film thickness was determined for SA/P/G and SA/P/G/PPE films using a digital micrometer (Mitutoyo, Japan) with a measurement precision of 0.01 mm, and the reported value represents the average of 10 randomly chosen locations on each film.

Mechanical characteristics of SA/P/G and SA/P/G/PPE films, including tensile strength (TS), elongation at break (EAB, %), and elastic modulus (EM), were determined using a TA.XT Plus texture analyzer (Stable Microsystems Ltd., UK) according to the ASTM D882 standard test method.^27^ Strips of films measuring 75 mm × 25 mm were prepared, conditioned at 25 °C and 50% relative humidity for 48 h, clamped with a 50 N initial force, and stretched at 100 mm/min over a 50 mm gauge length until breakage.

#### 2.2.4 MC (%) and WS (%)

2 cm × 2 cm sections of SA/P/G and SA/P/G/PPE films were weighed (*m*_1_), oven-dried at 105 °C for 24 h, reweighed (*m*_2_), and MC (%) was subsequently determined according to eq 1.

1 $$\mathrm{MC}\left(\%\right)=\frac{m_{1}-m_{2}}{m_{1}}\times 100$$

WS (%) was determined by weighing the dried film sample (*m*_3_, oven-dried at 60 °C overnight), immersing it in distilled water for under 1 min with gentle shaking, drying at 105 °C for 24 h to record the final weight (*m*_4_), and calculating WS (%) using eq 2 as shown below.

2 $$\mathrm{WS}\left(\%\right)=\frac{m_{3}-m_{4}}{m_{3}}\times 100$$

#### 2.2.5 WVP

The WVP of SA/P/G and SA/P/G/PPE films was measured gravimetrically following ASTM E96/E96M, with the films cut to appropriate sizes and placed over 5.8 cm diameter glass test containers.^28^ The glass test containers were loaded with approximately 10 g of CaCl_2_, exhibiting a nominal MC of 0%. Thereafter, the containers were positioned in a desiccator containing a saturated sodium chloride (NaCl) solution, maintaining 75% relative humidity at room temperature over 8 h, and the vials were weighed hourly to monitor changes in mass. The WVP of SA/P/G and SA/P/G/PPE film samples was determined using eq 3, and all measurements were performed in triplicate.

3 $$WVP\left(\frac{g}{m.s.Pa}\right)=\frac{\Delta m\times x}{A\times t\times \Delta P}$$

In the above equation, Δ*m* denotes the change in mass of the test containers in grams; *x* denotes the mean film thickness; *A* represents the surface area of the test film sample in m^2^; *t* represents the measurement duration, expressed in seconds; and Δ*P* signifies the differential water vapor pressure across the film at 25 °C.

#### 2.2.6 Color Evaluation

The color attributes of SA/P/G and SA/P/G/PPE films were measured using a digital colorimeter, preceded by calibration against a white standard plate. Film samples were placed on the same plate to measure L*, a*, and b* values, and the overall color difference (ΔE*) was determined using eq 4, where ΔL*, Δa*, and Δb* represent the deviations in lightness, redness-greenness, and yellowness-blueness relative to a white reference plate, respectively.

4 $${\Delta E}^{*}=\sqrt{{\left(\Delta L^{*}\right)}^{2}+{\left(\Delta a^{*}\right)}^{2}+{\left(\Delta b^{*}\right)}^{2}}$$

#### 2.2.7 Light Transmittance and Transparency

SA/P/G and SA/P/G/PPE film samples were sectioned into rectangular pieces of 4 cm × 1 cm to fit a quartz cuvette, after which their UV–Vis transmittance was measured over 200–800 nm using a spectrophotometer, with an empty cuvette serving as the blank. Film transparency was determined at 600 nm and subsequently calculated according to eq 5.

5 $$\mathrm{Transparency}=\frac{A_{600}}{x}$$

In the above equation, *A*_600_ represents the absorbance measured at 600 nm and *x* denotes the thickness in millimeters.

#### 2.2.8 Fourier Transform Infrared (FTIR) Spectroscopy

The interactions among components of SA/P/G films containing PPE were investigated using a PerkinElmer Spectrum 100 FTIR spectrometer. FTIR spectra were acquired for film specimens of 2 cm^2^, employing an ATR cell, with the samples placed in crystal holders, after which they were analyzed over 4000–500 cm^–1^ using 32 scans at a 2 cm^–1^ resolution.

#### 2.2.9 Scanning Electron Microscopy (SEM)

Microstructural features and morphology of SA/P/G- and SA/P/G/PPE-based films were analyzed via scanning electron microscopy (SEM) after cryo-fracturing the samples immersed in liquid nitrogen. The specimens were placed on metal holders, gold-coated by using a sputter coater, and subsequently imaged at various magnifications using a 10 kV accelerating voltage.

#### 2.2.10 Differential Scanning Calorimetry (DSC)

Film samples were subjected to DSC measurements employing a DSC Q20 thermal analyzer (TA Instruments). SA/P/G and SA/P/G/PPE film specimens (5–10 mg) were enclosed in aluminum pans and analyzed using DSC from 25 to 200 °C at a heating rate of 10 °C/min in a nitrogen atmosphere with constant purge flow.

#### 2.2.11 Antioxidant Properties of SA30/P30/G40/PPE Films

The antioxidant activity of the prepared SA30/P30/G40/PPE films was evaluated by measuring DPPH radical-scavenging activity using a modified method based on the standard DPPH assay reported by Patil et al.^29^ Briefly, film samples (2.5 g) were extracted with 17.5 mL of methanol, subjected to homogenization–stirring at 25 °C, filtered through Whatman No. 1 filter paper, and 0.1 mL of the obtained extract was mixed with 3.9 mL of DPPH solution (3.94 mg DPPH/100 mL methanol) for antioxidant activity determination. The mixture was vortexed vigorously and incubated in the dark for 30 min at 25 °C, after which the absorbance was measured at 517 nm using a UV/vis spectrophotometer. The antioxidant activity was expressed as DPPH radical-scavenging activity (%) and calculated using eq 6, with all measurements performed in triplicate, where *A*_(blank)_ and *A*_(sample)_ represent the absorbance values of the control and film extract samples, respectively.

6 $$\mathrm{DPPH}\left(\%\right)=\frac{A_{\mathrm{blank}}-A_{\mathrm{sample}}}{A_{\mathrm{blank}}}\times 100$$

The ABTS radical-scavenging activity of the films was determined according to the method of Zhao et al.^18^ with slight modifications. ABTS radical cation was prepared by mixing 7.2 mM ABTS with 2.6 mM potassium persulfate and storing the mixture in the dark at 25 °C for 14 h, after which it was diluted with ethanol to reach an absorbance of 0.70 ± 0.02 at 734 nm for further analyses. Each film sample (25 mg) was dissolved in 5 mL of ethanol for extraction, and 1.2 mL of the extract was mixed with 4.8 mL of the ABTS working solution, thoroughly shaken, incubated in the dark at room temperature for 6 min, and then measured at 734 nm using a UV–vis spectrophotometer with 95% (w/v) ethanol as the blank. The percentage of radical scavenging was calculated using eq 7, where *A*_blank_ and *A*_sample_ are the absorbances of the control and the sample, respectively, with all tests performed in triplicate.

7 $$ABTS\left(\%\right)=\frac{A_{blank}-A_{sample}}{A_{blank}}\times 100$$

#### 2.2.12 Statistical Analysis

The experimental data were evaluated using one-way ANOVA in SPSS 25.0 (Chicago, IL), with Duncan’s multiple range test for assessing significant differences (*p* < 0.05), and results are presented as mean ± standard deviation.

## 3 Results and Discussion

### 3.1 Characterization of SA/P/G-Based Films

#### 3.1.1 Thickness

Film thickness is a critical parameter in packaging applications, as it significantly influences mechanical strength as well as barrier, water vapor, UV, and optical properties of the material.^24,30^ Table 2 presents the thickness of SA/P/G films with varying ratios, revealing that increasing G content from 20% to 50% significantly increased thickness from 0.0732 mm (SA40/P40/G20) to 0.1328 mm (SA25/P25/G50), an approximate 81.3% rise (*p* < 0.05).

**Table 2:** Thickness, WS (%), MC (%), and WVP of Films Based on SA/P/G^a^

film samples	thickness (mm)	WS (%)	MC (%)	WVP (g mm/m^2^h kPa)
SA40/P40/G20	0.0732 ± 0.0071^d^	78.755 ± 2.377^a^	20.255 ± 0.417^a^	0.707 ± 0.385^a^
SA35/P35/G30	0.0849 ± 0.0062^c^	71.717 ± 2.562^b^	19.552 ± 0.485^b^	0.674 ± 0.242^b^
SA30/P30/G40	0.1180 ± 0.0095^b^	59.182 ± 2.020^d^	15.815 ± 0.822^d^	0.466 ± 0.097^d^
SA25/P25/G50	0.1328 ± 0.0101^a^	65.825 ± 1.980^c^	16.772 ± 0.292^c^	0.556 ± 0.190^c^

a Distinct letters within a single column denote statistically significant differences between film formulations as determined by Duncan’s test (*p* < 0.05).

Denavi et al.^31^ observed that, under identical protein content and drying conditions, G-containing films were thicker despite lower water content, indicating greater matrix compaction and differences in protein unfolding or cross-linking within the network. Chen et al.^32^ reported a thickness of 0.065 mm for the control SA/G film, while Hoque et al.^33^ observed a slightly higher thickness of 0.069 mm for P/SA films designed for use in food packaging.

#### 3.1.2 WS (%)

WS (%) reflects the hydrophilicity of biopolymer-based films and is a key parameter in determining application suitability, as low WS is generally preferred to preserve packaged food integrity, enhance moisture barrier performance, and extend shelf life.^34,35^ As presented in Table 2, WS (%) of SA/P/G films decreased from 78.755% (SA40/P40/G20) to 59.182% (SA30/P30/G40), corresponding to an approximately 24.9% reduction; WS declined significantly with increasing G content up to 40%, but increased at 50% G (*p* < 0.05), suggesting that moderate G reduced water affinity while excessive amounts disrupted the polymer network and enhanced water uptake. The incorporation of fish G enhanced the water resistance of chitosan/poly­(vinyl alcohol)/fish G ternary films, likely due to electrostatic interactions and hydrogen bonding that reduced free hydroxyl groups and limited polymer–water interactions, thereby decreasing solubility.^36^ Liu et al.^37^ reported that soluble soybean polysaccharide/G (80/20) films were completely water-soluble, whereas increasing G content to 40% markedly improved water resistance; overall, higher G fractions progressively reduced WS (%), accompanied by decreases in MC (%) and WVP, likely due to the formation of a more compact polymer matrix with reduced free volume. Varughese et al.^38^ determined the WS of P/SA films as 91.60%, whereas Elhadef et al.^35^ observed a lower WS of 75.10% for G/SA–based films. Fiallos-Nunez et al.^39^ reported that low-density polyethylene (LDPE) is widely used for food packaging due to its excellent barrier and mechanical properties, with WS values of 1.8% for LDPE and 24.3% for chitosan/G films.

#### 3.1.3 MC (%)

MC (%) reflects the free volume of water in a hygroscopic film matrix, where higher MC indicates a looser microstructure, and controlling MC is crucial for designing selective barriers that inhibit microbial growth and extend food shelf life.^40,41^ The MC (%) values of SA/P/G films with varying ratios are presented in Table 2, showing that the SA40/P40/G20 film exhibited the highest MC at 20.255%, whereas the SA30/P30/G40 film showed the lowest value at 15.815%, corresponding to an approximate decrease of 21.9%. In SA/P/G ternary film formulations, the MC (%) significantly decreased as G content increased up to 40%, but a further increase to 50% G led to a notable rise in MC (%) (*p* < 0.05). Esteves et al.^42^ reported that increasing G concentration in G–carboxymethyl cellulose films promoted hydrogen-bond interactions that reduced the accessibility of carboxyl and hydroxyl groups to water, thereby decreasing the films’ MC (%). In fish G films, gellan gum creates a more compact network that limits free water, reducing MC (%), while the matrix stabilized by hydrogen bonds, hydrophobic interactions, and van der Waals forces enhances water retention capacity.^43^ Bhatia et al.^44^ reported an MC of 22.28% for P/SA-based edible films, while Varughese et al.^38^ observed a slightly lower MC of 20.32% for P/SA-based films.

#### 3.1.4 WVP

In food packaging applications, the diffusion of water molecules through the film matrix is defined as WVP, a critical parameter because low WVP values reduce moisture transfer, limit microbial growth, and extend shelf life, while barrier performance against water vapor is also influenced by environmental conditions, film structure, and the physicochemical properties of the biopolymers used.^41,45^ As shown in Table 2, WVP of SA/P/G films ranged from 0.707 g·mm/m^2^ h kPa (SA40/P40/G20) to 0.4.66 g·mm/m^2^ h kPa (SA30/P30/G40), representing an approximate 34% decrease; WVP decreased significantly with G content up to 40% but increased at 50% G (*p* < 0.05). Increasing G concentration up to 40% fills cavities and molecular gaps within the film network, enhancing density and reducing porosity, thereby lowering WVP and producing a more compact matrix in SA30/P30/G40 than in SA40/P40/G20; however, at 50% G content, a significant increase in WVP was observed again, consistent with SEM results. Pectin–G blend films exhibited a WVP of 6.57 g·mm/m^2^·h·kPa,^46^ amphotericin B-loaded SA/carboxymethyl cellulose/G-based films showed WVP values of 0.187–0.334 g·mm/m^2^·h·kPa,^47^ and fish G–glycerol films demonstrated a WVP of 11.52 g·mm/m^2^·d·kPa.^48^

On the basis of these findings, the SA30/P30/G40 formulation, exhibiting the lowest MC (%), WS (%), and WVP, was selected to investigate the effects of varying PPE concentrations on the physical, mechanical, and barrier characteristics of the resulting films.

### 3.2 Characterization of SA30/P30/G40 and SA30/P30/G40/PPE-Based Films

#### 3.2.1 Thickness

Table 3 presents the thickness of SA30/P30/G40 films with varying PPE concentrations, showing that the control film exhibited the minimum thickness (0.118 mm), which progressively increased to 0.146 mm at 2% PPE—an approximate 23.7% rise—with all PPE-containing films significantly thicker than the control (*p* < 0.05). Hydrogen-bonding-mediated cross-linking between biopolymer chains and high-molecular-weight phenolic compounds in PPE, along with increased solid content, drives the increase in film thickness, reflecting the interaction of polyphenolic bioactive compounds with the polymer matrix.^17,23^

**Table 3:** Thickness, WS (%), MC (%), and WVP Values of SA30/P30/G40 and SA30/P30/G40-PPE-Based Films^a^

film samples	thickness (mm)	WS (%)	MC (%)	WVP (g·mm/m^2^h kPa)
SA30/P30/G40	0.118 ± 0.009^e^	59.182 ± 2.020^a^	15.815 ± 0.822^a^	0.466 ± 0.097^a^
SA30/P30/G40/PPE^%0.5^	0.129 ± 0.004^d^	53.157 ± 2.919^b^	13.149 ± 1.342^b^	0.369 ± 0.075^b^
SA30/P30/G40/PPE^%1^	0.134 ± 0.003^c^	45.765 ± 2.808^c^	11.133 ± 0.923^c^	0.313 ± 0.055^c^
SA30/P30/G40/PPE^%1.5^	0.138 ± 0.006^b^	41.637 ± 2.699^d^	9.972 ± 0.873^d^	0.278 ± 0.047^d^
SA30/P30/G40/PPE^%2^	0.146 ± 0.004^a^	40.047 ± 3.089^d^	9.702 ± 1.019^d^	0.258 ± 0.051^e^

a Distinct letters within a single column denote statistically significant differences between film formulations as determined by Duncan’s test (*p* < 0.05).

The effects of PPE on film thickness have been studied in various polymer matrices: Hanani et al.^15^ reported that increasing PPE content from 1 to 2% in fish gelatin (G)-based films substantially increased film thickness (*p* < 0.05), attributed to the incorporation of PP powder containing a combination of soluble and insoluble fibers; similarly, Ahmad et al.^23^ found that low PPE incorporation (0.5%) had no significant impact on the thickness of carboxymethyl cellulose/G films, whereas higher concentrations (1–2%) caused a significant increase, in line with Vargas-Torrico et al.,^24^ which linked the increase in thickness at elevated PPE levels to increased solid content and phenolic compound incorporation into the polymer matrix.

#### 3.2.2 WS (%)

The WS (%) values of SA30/P30/G40 and SA30/P30/G40 films with varying PPE concentrations (Table 3) indicated that the control film displayed the highest WS (59.182%), while the lowest value was observed at 2% PPE (40.047%), representing an overall reduction of approximately 32.3% and a concentration-dependent decrease compared to the control. Pomegranate-derived powders or extracts have been reported to substantially reduce the WS of biopolymer-based films, with fish G films decreasing from 68.64 to 54.59%,^15^ G/chitosan composites from 50.74 to 19.80%,^49^ and G/carboxymethyl cellulose films from 52.67 to 33.81%,^24^ due to phenolic- and sugar-mediated cross-linking, protein aggregation, α-helix-to-β-sheet transitions, disruption of hydrogen-bonded networks, reduced hydroxyl–water interactions, and the formation of denser, more hydrophobic polymer matrices.

#### 3.2.3 MC (%)

Film water sensitivity, especially MC (%), is critical for predicting performance, as it affects flexibility, durability, and the shelf life of packaged food.^24,50^ The MC (%) values of SA30/P30/G40-based films with varying PPE concentrations are shown in Table 3, with the control film exhibiting the highest MC (15.815%) due to component affinity and hydrogen-bonded networks, while films containing 2% PPE showed the lowest MC (9.702%), a reduction of ∼ 38.6%, and MC decreased significantly (*p* < 0.05) as PPE content increased up to 2% compared to the control. MC (%) of biopolymer-based films varies in a matrix-dependent manner, with fish G films showing minimal change, 11.05–10.33%^15^ due to balanced hydrophilic–hydrophobic constituents. Hajirostamloo et al.^22^ reported soy protein isolate/seed gum films containing a PPE–carrot seed extract mixture decreasing from 25.36 to 22.44%, and G/carboxymethyl cellulose films exhibiting significant reductions, 31.13 to 18.78% from extract-induced interactions.^24^

#### 3.2.4 WVP

WVP, which refers to the rate at which water vapor passes through a material, is a crucial factor in film development because it regulates moisture transfer, affecting both product quality and shelf life; decreasing WVP in polymer-based films helps preserve the MC of the products being packaged.^15,23,24^ Table 3 shows the WVP of SA30/P30/G40 films with varying PPE concentrations, with the highest value for the control sample (0.466 g·mm/m^2^ h kPa) and the lowest in the 2% PPE film (0.258 g·mm/m^2^ h kPa), representing an overall decrease of approximately 44.6%. Consistent with SEM observations, increasing PPE concentration in SA30/P30/G40 films filled intermediate spaces, producing denser and more homogeneous structures, while Hajirostamloo et al.^22^ reported that WVP of soy protein isolate/seed gum films decreased from 2.01 to 0.61 g·mm/m^2^·d·kPa as the proportion of PPE–carrot seed extract increased, highlighting the effect of extract incorporation on reducing WVP. Modifications in WVP of silver carp surimi-based films enriched with PPE can be attributed to differences in hydrophobicity, the formation of complex networks, and the cross-linking interactions between proteins and phenolic compounds.^51^

#### 3.2.5 Mechanical Properties

The mechanical characteristics, particularly TS and EAB (%), serve as primary measures of a film’s capacity to preserve the physical condition of products across storage, distribution, and commercialization stages.^15,23,24^

Table 4 presents the effect of different PPE concentrations on the mechanical behavior of the SA30/P30/G40 films.

**Table 4:** TS, EAB (%), and EM of SA30/P30/G40 Films with Varying PPE Concentrations^a^

film samples	TS (MPa)	EAB (%)	EM (MPa)	DPPH (%)	ABTS (%)
SA30/P30/G40	14.201 ± 1.064^e^	57.049 ± 3.433^a^	251.997 ± 9.645^e^	10.547 ± 0.475^e^	14.373 ± 0.324^e^
SA30/P30/G40/PPE^%0.5^	17.959 ± 1.530^d^	49.090 ± 2.890^b^	312.561 ± 11.755^d^	28.447 ± 1.182^d^	19.202 ± 0.505^d^
SA30/P30/G40/PPE^%1^	21.927 ± 0.854^c^	44.027 ± 2.138^c^	354.133 ± 1.973^c^	47.965 ± 1.220^c^	39.261 ± 0.567^c^
SA30/P30/G40/PPE^%1.5^	25.721 ± 2.443^b^	41.272 ± 2.928^d^	407.195 ± 10.635^b^	64.667 ± 1.140^b^	55.984 ± 0.417^b^
SA30/P30/G40/PPE^%2^	29.209 ± 1.766^a^	37.167 ± 2.361^e^	471.065 ± 18.080^a^	85.635 ± 0.115^a^	78.760 ± 1.269^a^

a Distinct letters within a single column denote statistically significant differences between film formulations as determined by Duncan’s test (*p* < 0.05).

The control SA30/P30/G40-based film exhibited the lowest TS (14.201 MPa), whereas incorporation of PPE at varying concentrations, notably 2%, significantly (*p* < 0.05) enhanced TS up to 29.209 MPa, indicating an overall rise of about 105.7% compared with the control. The increased TS values of the films can be attributed to the reinforcing effect of homogeneously distributed PPE within the SA30/P30/G40 matrix, where polyphenols in PPE formed hydrogen bonds with protein carboxyl groups, enhancing intermolecular interactions and strengthening the polymer network against mechanical stress.^18^ Fish G films with 1–5% PP powder^15^ exhibited TS increases up to 8.02 MPa, whereas P/SA/whey protein concentrate-based films^52^ had a TS of 23 MPa, pectin films^53^ 59.2 MPa, fish skin G films cross-linked with 0.1% glutaraldehyde^53^ 99.2 MPa, and carboxymethyl cellulose/G films with PPE^23^ showed enhanced TS via phenolic-induced hydrogen bonding. Tsai et al.^54^ reported that LDPE film exhibits a TS of 18.96 MPa, whereas Sulistiawan et al.^56^ reported a TS of 17.9 MPa.

EAB (%) measures a film’s stretchability and reflects its capacity to deform under applied stress.^15^ SA30/P30/G40-based films showed the EAB of 57.049% without PPE and the lowest of 37.167% with 2% PPE, representing an overall decrease of approximately 34.9%, with all PPE-containing films exhibiting a significant (*p* < 0.05) reduction in EAB relative to the control. The addition of PPE reduced the EAB of the films by promoting cross-linking and stronger intermolecular interactions within the polymer matrix, which restricted chain mobility and simultaneously improved water barrier properties at higher PPE concentrations. LiuLiu et al.^53^ observed that fish skin G films cross-linked with 0.1% glutaraldehyde exhibited an EAB of 3.6%, whereas pectin films showed 1.7%, indicating polysaccharide brittleness. Chakravartula et al.^52^ found that P/SA/whey protein films had an intermediate EAB of 22%, reflecting enhanced flexibility. Hanani et al.^15^ reported that fish G films with PP powder exhibited low EAB (2.09%) with no significant change upon increasing PP powder. Ahmad et al.^23^ observed that carboxymethyl cellulose/G films with PPE initially increased EAB to 46.167% at 0.5%, then decreased to 29.474% at 1.5%, showing concentration-dependent effects of phenolic compounds on film extensibility. Thongpool et al.^55^ reported that LDPE film exhibits EAB of 394.76%, whereas Sulistiawan et al.^56^ reported an EAB of 188.2%.

SA30/P30/G40-based films exhibited the lowest EM (251.997 MPa) without PPE and the highest (471.065 MPa) with 2% PPE, corresponding to an overall increase of approximately 86.9%, with all PPE-containing films showing a statistically significant (*p* < 0.05) improvement in EM compared to the control. P/SA/whey protein concentrate-based composite edible films exhibited an EM of 444 MPa.^52^

#### 3.2.6 Color

Film color critically modulates physicochemical appearance and consumer perception, with chromatic attributes in active biopolymer-based matrices predominantly dictated by the nature, origin, and molecular composition of incorporated pigmenting agents, underscoring its pivotal role in sensory acceptability and visual quality; furthermore, film appearance and color in food packages are key factors strongly influencing buyer acceptance of packaging materials.^24,57,58^

Table 5 presents the L*, a*, b*, and ΔE* color parameters of SA30/P30/G40 films with varying PPE concentrations.

**Table 5:** Color Values of SA30/P30/G40-Based Films with Varying PPE Concentrations

film samples	*L* ^a^	*a* ^a^	*b* ^a^	Δ*E* ^a^
SA30/P30/G40	86.986 ± 0.198^a^	–1.565 ± 0.148^e^	5.506 ± 0.180^e^	8.68 ± 0.16^e^
SA30/P30/G40/PPE^%0.5^	82.463 ± 0.303^b^	0.127 ± 0.355^d^	7.721 ± 0.301^d^	14.967 ± 0.195^d^
SA30/P30/G40/PPE^%1^	78.222 ± 0.428^c^	1.033 ± 0.297^c^	9.220 ± 0.291^c^	19.927 ± 0.140^c^
SA30/P30/G40/PPE^%1.5^	76.548 ± 0.447^d^	2.260 ± 0.245^b^	13.831 ± 0.313^b^	22.508 ± 0.355^b^
SA30/P30/G40/PPE^%2^	73.084 ± 0.403^e^	3.463 ± 0.227^a^	15.309 ± 0.326^a^	26.492 ± 0.835^a^

a Distinct letters within a single column denote statistically significant differences between film formulations as determined by Duncan’s test (*p* < 0.05).

SA30/P30/G40-based films showed the highest L* value (86.986) without PPE and the lowest (73.084) at 2% PPE, corresponding to an approximate 16% decrease, with all PPE-containing films exhibiting a significant (*p* < 0.05) decrease relative to the control; similarly, carboxymethyl cellulose/G-based films,^23^ fish G-based films,^15^ taro starch–casein films,^57^ and starch films^58^ all exhibited concentration-dependent decreases in L* upon PPE or PP powder incorporation.

The SA30/P30/G40-based control film exhibited the lowest a* value (−1.565), whereas PPE incorporation up to 2% resulted in the maximum a* coordinate (3.463), corresponding to an approximately 321% increase in redness index, with all PPE-containing films showing a statistically significant (*p* < 0.05) elevation in a* values relative to the control; in agreement, Hanani et al.^15^ and de Almeida Soares and de Aquino Santana^59^ reported concentration-dependent increases in a* in PP-enriched G- and chitosan-based films, attributing this effect to anthocyanins and other phenolic compounds responsible for red-purple pigmentation, while Esfahani et al.^58^ observed that starch- and PPE-based films exhibited the lowest a* (1.05) and that redness increased significantly (*p* < 0.05), reaching the highest a* (13.67) in films containing the maximum PPE concentration.

The SA30/P30/G40-based control film exhibited the minimum b* value (5.506), whereas PPE incorporation up to 2% resulted in the maximum b* coordinate (15.309), corresponding to an approximately 178% increase in yellowness index, with all PPE-containing films showing a statistically significant elevation in b* relative to the control (*p* < 0.05); consistent with these findings, Ahmad et al.^23^ observed a marked increase in b* values in carboxymethyl cellulose/G-based films with PPE addition, de Almeida Soares and de Aquino Santana^59^ reported a concentration-dependent rise in b* in biodegradable chitosan-based films, and Hanani et al.^15^ demonstrated that increasing PP powder content in fish G-based films significantly enhanced film yellowness (*p* < 0.05), an effect attributed to phenolic compounds and anthocyanin pigments; similarly, Esfahani et al.^58^ reported that starch- and PPE-based films exhibited the lowest b* value (3.39) and that yellowness increased significantly (*p* < 0.05), reaching the highest b* value (17.15) in films containing the maximum PPE concentration.

The lowest ΔE* value was observed in the extract-free SA30/P30/G40-based film (8.68), while the highest ΔE* value occurred in the film containing 2% PPE (26.492), representing an approximately 205% increase; accordingly, PPE-containing films showed a statistically significant elevation in ΔE* relative to the control (*p* < 0.05), indicating enhanced overall color change with extract incorporation. In agreement, Esfahani et al.^58^ observed a significant increase in ΔE* in starch- and PPE-based films, with the highest ΔE* (20.58) observed at the maximum PPE level, while Ahmad et al.^23^ observed that increasing PPE concentration in carboxymethyl cellulose/G-based films significantly raised ΔE* from 6.98 in the control to 60.16 at the highest extract level.

#### 3.2.7 Light Transmittance and Transparency

Light transmittance critically affects edible films, influencing both visual appearance and light-induced reactions such as lipid oxidation, while films with strong UV barrier properties prevent quality deterioration; light barrier capacity is essential for packaging effectiveness, as UV absorption can promote lipid oxidation and impair food quality.^60–63^ Table 6 presents the UV and visible light transmittance values measured at specific wavelengths (200–800 nm) for the control and SA30/P30/G40/PPE-based films containing different concentrations of PPE. Under UV light, SA30/P30/G40 films exhibited low transmittance, particularly at 200 and 280 nm, due to the pronounced UV-absorbing properties of aromatic amino acids like phenylalanine and tyrosine in G.^60^ Adding PPE markedly decreased the light transmittance of SA30/P30/G40-based films across all UV wavelengths, demonstrating a strong UV-blocking capacity that increased progressively with higher extract concentrations. Studies have shown that adding phenolic-rich extracts improves the UV-blocking ability of G-based films: Dou et al.^61^ showed that G/SA films demonstrated markedly lower transmittance in the 200–280 nm UV range, with transmittance at 280 nm decreasing from 59.02 to 2.22% as tea polyphenol content increased due to n→π* transitions in polyphenol benzene rings, while Getachew et al.^62^ reported that fish skin G films containing spent coffee grounds extract exhibited improved UV absorption attributed to phenolic and flavonoid compounds with benzene, −OH, and C=O groups. UV light transmittance decreases due to aromatic structures: epigallocatechin gallate-incorporated PLA/chitosan–G films show zero transmittance at 200 nm owing to aromatic rings and unsaturated bonds, while tyrosine and phenylalanine residues in fish gelatin polypeptides similarly absorb light and hinder optical transmission.^64^ Vargas-Torrico et al.^24^ observed that G/carboxymethyl cellulose films containing PPE exhibited effective UV–vis light-blocking properties due to UV–vis-absorbing polyphenolic pigments such as ellagic acid and punicalagin, regardless of extract concentration.

**Table 6:** Light Transmittance and Transparency of SA30/P30/G40 and SA30/P30/G40 Films Containing Varying Concentrations of PPE^a^

	light transmittance (%) at different wavelengths (nm)								
film samples	200	280	350	400	500	600	700	800	transparency
SA30/P30/G40	0.295 ± 0.042	42.807 ± 0.580	52.135 ± 1.227	62.360 ± 1.021	71.550 ± 1.09	76.525 ± 0.519	81.119 ± 0.947	84.746 ± 0.743	1.173 ± 0.024^e^
SA30/P30/G40/PPE^%0.5^	0.241 ± 0.023	12.346 ± 0.685	42.077 ± 1.143	56.455 ± 1.050	67.296 ± 0.975	72.665 ± 0.94	78.233 ± 0.584	82.061 ± 0.905	1.455 ± 0.030^d^
SA30/P30/G40/PPE^%1^	0.228 ± 0.019	5.888 ± 0.740	37.875 ± 1.262	53.036 ± 0.684	63.374 ± 0.781	69.676 ± 0.923	76.255 ± 0.840	80.026 ± 0.862	1.761 ± 0.073^c^
SA30/P30/G40/PPE^%1.5^	0.222 ± 0.037	3.759 ± 0.133	34.958 ± 0.693	50.951 ± 0.665	60.150 ± 0.609	66.575 ± 1.455	72.767 ± 0.737	78.453 ± 1.239	1.927 ± 0.040^b^
SA30/P30/G40/PPE^%2^	0.173 ± 0.073	2.031 ± 0.069	32.243 ± 0.546	47.777 ± 1.014	56.985 ± 1.048	62.910 ± 0.619	68.414 ± 1.841	74.734 ± 1.101	2.134 ± 0.076^a^

a Distinct letters within a single column denote statistically significant differences between film formulations as determined by Duncan’s test (*p* < 0.05).

The transparency of a film is a critical factor influencing its potential applications.^61^ The lowest transparency was observed in the extract-free SA30/P30/G40-based film (1.173), while the highest transparency occurred in the film containing 2% PPE (2.134), corresponding to an approximately 82% increase; furthermore, PPE-containing films demonstrated a notable (*p* < 0.05) rise in transparency relative to the control. Previous studies consistently demonstrate that adding polyphenol-enriched extracts alters the optical properties of biopolymer-based films. Tea polyphenols in G/SA films^61^ and spent coffee grounds extract in fish skin G films^62^ increased transparency indices while simultaneously enhancing opacity. In contrast, PPE incorporation reduced optical transparency and light transmittance in taro starch–casein films^57^ and in G–carboxymethyl cellulose films,^23,24^ leading to significantly higher opacity.

#### 3.2.8 FTIR

To examine the chemical interactions among film components and structural changes induced by varying PPE concentrations, FTIR spectroscopy was employed, with the resulting spectra in the 500–4000 cm^–1^ range presented in Figure 1.

**Figure 1:**
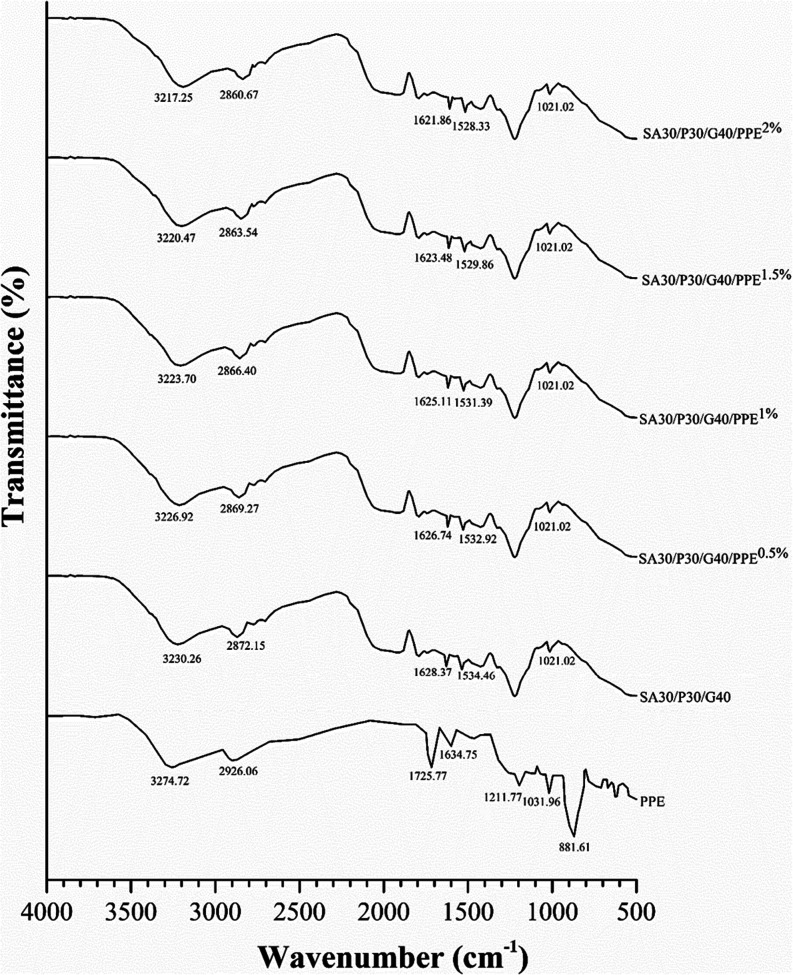
FTIR spectra of SA30/P30/G40-based films containing PPE at different concentrations.

Figure 1 shows that all SA/P/G-based films exhibit a sharp peak at 1021.02 cm^–1^, indicating the effective integration of glycerol as a plasticizer, corresponding to the O–H functional group of glycerol.^23^ Shifts in band wavenumbers were observed in SA30/P30/G40 films with and without PPE, probably resulting from hydrogen bonding and electrostatic interactions between SA30/P30/G40 and extract compounds.^59^ As shown in Figure 1, the characteristic bands of PPE were observed at 3274.72, 2929.06, 1725.77, 1634.75, 1211.77, 1031.96, and 881.61 cm^–1^, corresponding to vibrational stretching of O–H groups (including aromatic ring hydroxyls), aromatic C–H bonds, carbonyl (C=O) groups, C=C bonds, epoxide (C–O–C) groups, and C–O bonds.^24,65–67^ The amide A peak observed at 3230.26 cm^–1^ in SA30/P30/G40-based film samples indicates the existence of unbound O–H and N–H groups.^68^ Figure 1 shows that the vibrational band attributed to the −CH_3_ group exhibited a shift from 2872.15 to 2869.27 cm^–1^, indicating alterations in the molecular environment within the film matrix.^23,66,68^ The addition of varying PPE concentrations caused shifts in amide II (involving N–H bending and C–N stretching) and amide I (involving C=O stretching) bands from 1534.46 to 1532.92 cm^–1^ and from 1628.37 to 1626.74 cm^–1^, respectively, with amide I being the key peak for evaluating the secondary structure of proteins by FTIR.^23,24,66,68^ These findings indicate that PPE induces alterations in the molecular organization of the SA30/P30/G40-based film matrix.^24^ As illustrated in Figure 1, adding PPE caused noticeable shifts for both of these characteristic signals. This displacement of peak locations could be attributed to the formation of H bonds inside the film network.^23^ The lack of new functional peaks following PPE incorporation indicates an absence of chemical reactions occurring between components within the film network (SA30/P30/G40).

#### 3.2.9 SEM

The interactions among the components in the film formulation determine the composition of the film samples.^69^ SEM was performed to characterize the topography and structural texture of SA30/P30/G30 films and those containing varying ratios of PPE, as shown in Figure 2. The SA30/P30/G40-based films containing PPE were observed to exhibit a more compact and dense cross-sectional morphology compared to the control film (Figure 2). Figure 2 shows that all film samples exhibited smooth, crack- and void-free surfaces, indicating good compatibility among SA, P, and G in the polymer matrix and effective interaction of PPE with the network.^24,70^ Vargas-Torrico et al.^24^ reported that G/carboxymethyl cellulose films were compact and homogeneous, with protein–polysaccharide components forming noncovalent interactions, while the incorporation of increasing PPE levels produced rough, layered surfaces proportional to extract content. Therefore, PPE can be considered an effective filler for protein–polysaccharide films, potentially improving the films’ mechanical strength and barrier performance.^57^ The addition of PPE was observed to enhance the homogeneity of SA30/P30/G30-based films (Figure 2). Control chitosan films exhibited smooth surfaces, whereas 9% PP powder induced grooves and particulates due to poor dispersion;^71^ similarly, control fish G films were homogeneous, while 30 wt% pomegranate seed juice byproduct increased surface heterogeneity,^72^ and soy protein isolate/*Alyssum homolocarpum* seed gum films with 1–5% PPE showed continuous, homogeneous structures, whereas 10–20% PPE caused nonhomogeneous structures due to protein–polyphenol interactions.^22^

**Figure 2:**
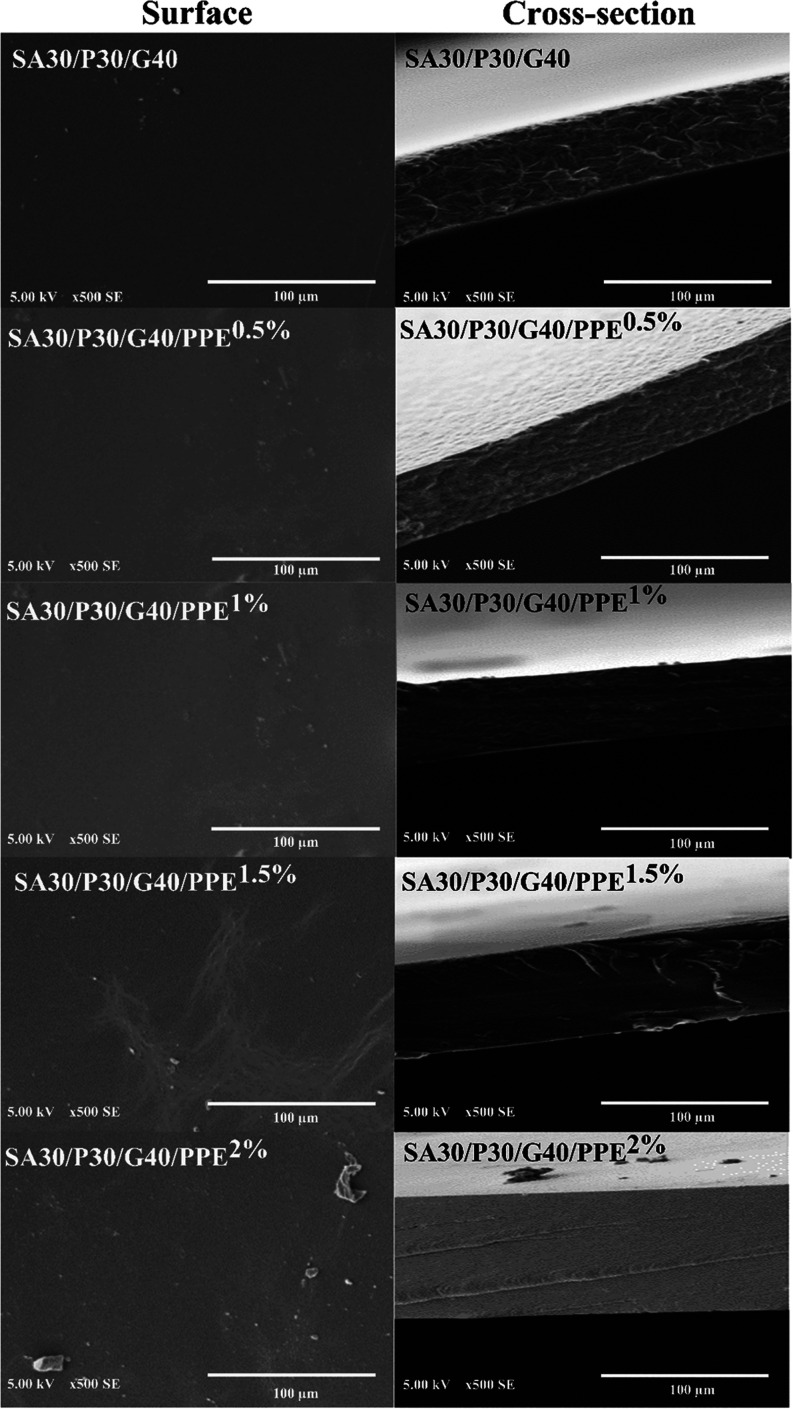
SEM micrographs of SA30/P30/G40 and SA30/P30/G40/PPE-based films with different PPE concentrations.

Examination of the cross-sectional images in Figure 2 reveals that all SA30/P30/G30-based films exhibit a reduction in small pores within their continuous structures, resulting in a more compact and dense morphology. Overall, compared with the control, PPE-enriched films exhibited a more continuous structure, attributed to interactions between protein/polysaccharide chains and extract polyphenols.^22^ SEM analyses revealed that low PP levels (2.5–3%) had minimal effects and ensured good compatibility in mung bean protein films (70), whereas higher PP (12.5–25%) caused voids and heterogeneity; similarly, chitosan films showed layered structures and agglomerations at 6–9% PP,^71^ and high-amylose corn starch/konjac glucomannan films with 1% PPE exhibited rough, cracked surfaces, while 2 wt% PPE produced homogeneous, dense surfaces via even distribution and cross-linking.^73^

#### 3.2.10 DSC

Examining structural changes in packaging materials under temperature variations is essential to assess the materials’ heat resistance.^74^ Thermal properties of both pure SA/P/G films and SA/P/G/PPE-based films incorporated with different amounts of PPE were examined using DSC, and the resulting thermograms are presented in Figure 3.

**Figure 3:**
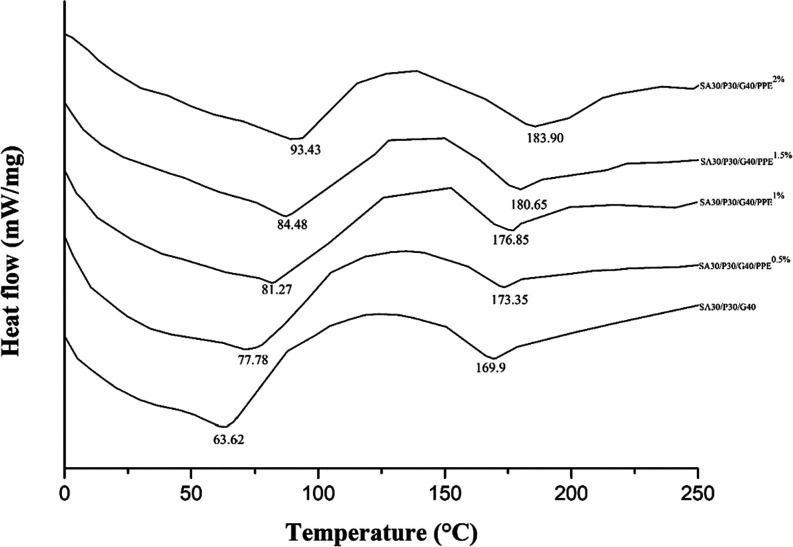
DSC curves of SA30/P30/G40 and SA30/P30/G40/PPE-based films containing varying concentrations of PPE.

The thermograms of the five displayed broad endothermic peaks in the regions corresponding to transitions or reactions occurring during DSC analysis. In the ALJ30/PKT30/JLT40 film, a broad endothermic peak appeared at 63.62 °C, reflecting the evaporation of volatile compounds, water, or chain relaxation events, while the endothermic peak temperatures of SA30/P30/G40 films increased to 93.43 °C with 2% PPE, representing an approximate 47% rise. In this context, increased chain rigidity and stronger intermolecular and intramolecular interactions, together with higher melting and degradation temperatures, indicate more ordered structures, enhanced barrier performance, and strengthened intermolecular interactions.^75,76^ Yu et al.^77^ reported that PVDF ultrafiltration membranes containing PP powder exhibited a single DSC peak, indicating good compatibility, while higher PP powder content increased melting temperature.

The second endothermic peaks are linked to the degradation within the film components’ structure.^76^ In the SA30/P30/G40 control film, the second endothermic peak appeared at 169.90 °C, while films containing 0.5–2% PPE exhibited a gradual increase from 173.35 to 183.90 °C, representing an approximate 6% rise in peak temperatures compared with the control. An increase in the degree of covalent cross-linking, accompanied by a decrease in the number of hydrogen bonds, contributes to enhanced thermal stability.^75^ It was observed that the structural integrity of the SA30/P30/G40 film network improved with the addition of PPE, with enhanced TS and EAB (%), and that these thermal behaviors were confirmed through SEM and mechanical property analyses.^76^ Similar to our findings, in chitosan,^76^ fish G,^75^ and G/chitosan-lactate^78^ films, incorporation of guava leaf extract, fructan, or turmeric extract, respectively, enhanced intermolecular and hydrogen bonding interactions, restricted polymer chain mobility, increased transition or melting temperatures, shifted endothermic peaks to higher values, improved thermal stability, and promoted a more compact film structure.

#### 3.2.11 Antioxidant Activity

The antioxidant activity of functional films, which contributes to delaying oxidative degradation and extending shelf life, is commonly evaluated using the standardized DPPH radical-scavenging assay based on the reduction of DPPH and the corresponding decrease in absorbance at 517 nm.^29,79^

PPE incorporation significantly (*p* < 0.05) improved the antioxidant activity of SA30/P30/G40-based films, increasing DPPH radical inhibition from 10.547% to a maximum of 85.635%, with the highest activity observed in the SA30/P30/G40/PPE^2%^ formulation, while the control activity was attributed to G (Table 4). The antioxidant activity of the control film was attributed to G due to its amino acids and peptides capable of donating electrons to neutralize free radicals, while the enhanced activity in PPE-containing films was associated with polyphenolic compounds such as ellagic acid, punicalagin, and gallic acid.^23^ Mung bean protein-based films enriched with PPE showed DPPH radical-scavenging activity ranging from 5.00 to 65.22%,^70^ while polyvinyl alcohol-based films containing nanokaolin impregnated with PPE exhibited values between 0.51 and 74.82%.^80^

The ABTS radical assay is a widely used, fast, practical, and stable method in which the blue-green ABTS radical cation, formed by ABTS oxidation, is reduced by antioxidants, leading to a solution discoloration.^15,81^ The results indicate that the control film without PPE has a relatively weak ABTS^+^ radical-scavenging capability, which increased significantly (*p* < 0.05) with higher PPE concentrations, demonstrating a marked improvement in antiradical activity. The control SA30/P30/G40-based film showed the lowest ABTS radical-scavenging activity (14.37%), while the incorporation of 2% PPE significantly (*p* < 0.05) increased activity to 78.76%, representing an overall rise of approximately 448% compared with the control. The increase in ABTS radical-scavenging activity is likely due to the high content of phenolic compounds in PPE, such as ellagic acid, punicalagin, and gallic acid, which are well known for their antioxidant properties, enhancing film performance.^18^ Dai et al.^25^ reported that polylactic acid films co-reinforced with PPE and nano-ZnO exhibited a maximum ABTS radical-scavenging activity of 93.1%, with activity increasing significantly at higher PPE concentrations, while Diaz-Herrera et al.^81^ found that pectin films containing 200 ppm of PPE inhibited ABTS free radicals by 48.5%.

## 4 Conclusion

The food and agriculture industries generate substantial waste, threatening sustainability. Recycling this waste into value-added products is increasingly important. In this study, SA/P/G-based films containing varying concentrations of PPE (0.5–2.0%) were prepared and characterized. Four formulations were initially tested, and the SA30/P30/G40 ratio was selected as optimal based on the lowest MC (%), WS (%), and WVP. The effects of different PPE concentrations on the films’ physical, mechanical, barrier, thermal, and morphological properties were then investigated. SA30/P30/G40-PPE films with 2% PPE showed improved mechanical and barrier properties, with TS increasing from 14.201 to 29.209 MPa, EM increasing from 251.997 to 471.065 MPa, WVP decreasing from 0.466 to 0.258 g·mm/m^2^ h kPa, MC decreasing from 15.815 to 9.702%, and WS decreasing from 59.182 to 40.047% relative to the control. PPE addition also reduced lightness, enhanced red and yellow tones, and increased opacity, lowering light transmittance. FTIR confirmed preservation of biopolymer functional groups, while SEM showed a smooth, crack-free surface with improved polymer compatibility. These findings highlight the potential of pomegranate byproducts for producing sustainable, cost-effective SA/P/G films with enhanced structural integrity, thermal stability, and surface smoothness, offering an environmentally friendly alternative to plastic packaging. Future studies should explore long-term stability, antimicrobial activity, and scalability, while addressing limitations such as environmental factors affecting film performance.
